# Response of Methanogens in Arctic Sediments to Temperature and Methanogenic Substrate Availability

**DOI:** 10.1371/journal.pone.0129733

**Published:** 2015-06-17

**Authors:** Lynsay I. Blake, Alexander Tveit, Lise Øvreås, Ian M. Head, Neil D. Gray

**Affiliations:** 1 Newcastle University, School of Civil engineering and Geosciences, Newcastle upon Tyne, United Kingdom; 2 Department of Arctic and Marine Biology, University of Tromsø, Tromsø, Norway; 3 Department of Biology and Centre for Geobiology, University of Bergen, Bergen, Norway; J. Craig Venter Institute, UNITED STATES

## Abstract

Although cold environments are major contributors to global biogeochemical cycles, comparatively little is known about their microbial community function, structure, and limits of activity. In this study a microcosm based approach was used to investigate the effects of temperature, and methanogenic substrate amendment, (acetate, methanol and H_2_/CO_2_) on methanogen activity and methanogen community structure in high Arctic wetlands (Solvatnet and Stuphallet, Svalbard). Methane production was not detected in Stuphallet sediment microcosms (over a 150 day period) and occurred within Solvatnet sediments microcosms (within 24 hours) at temperatures from 5 to 40°C, the maximum temperature being at far higher than *in situ* maximum temperatures (which range from air temperatures of -1.4 to 14.1°C during summer months). Distinct responses were observed in the Solvatnet methanogen community under different short term incubation conditions. Specifically, different communities were selected at higher and lower temperatures. At lower temperatures (5°C) addition of exogenous substrates (acetate, methanol or H_2_/CO_2_) had no stimulatory effect on the rate of methanogenesis or on methanogen community structure. The community in these incubations was dominated by members of the *Methanoregulaceae*/WCHA2-08 family-level group, which were most similar to the psychrotolerant hydrogenotrophic methanogen *Methanosphaerula palustris* strain E1-9c. In contrast, at higher temperatures, substrate amendment enhanced methane production in H_2_/CO_2_ amended microcosms, and played a clear role in structuring methanogen communities. Specifically, at 30°C members of the *Methanoregulaceae*/WCHA2-08 predominated following incubation with H_2_/CO_2_, and *Methanosarcinaceae*and *Methanosaetaceae* were enriched in response to acetate addition. These results may indicate that in transiently cold environments, methanogen communities can rapidly respond to moderate short term increases in temperature, but not necessarily to the seasonal release of previously frozen organic carbon from thawing permafrost soils. However, as temperatures increase such inputs of carbon will likely have a greater influence on methane production and methanogen community structure. Understanding the action and limitations of anaerobic microorganisms within cold environments may provide information which can be used in defining region-specific differences in the microbial processes; which ultimately control methane flux to the atmosphere.

## Introduction

In cold Arctic and sub-Arctic regions organic matter degradation is a slow process which has resulted in the accumulation of large quantities of organic matter within soils and sediments [1]. Nevertheless, Arctic and sub-Arctic regions contribute between 17 to 42 Tg of CH_4_ per annum to the global atmospheric methane flux (~25% of the global methane emissions from natural sources) [2–3]. It is anticipated that increased Arctic and sub-Arctic exposure to warmer winter periods will increase the frequency of winter snow melt/refreeze, enhance precipitation, and lead to a greater proportion of winter precipitation falling as rain [4–7]. These factors will potentially lead to elevated periods of flood-induced hypoxia, which along with increased ambient temperature, may stimulate anaerobic microbial degradation processes [8]. Consequently, the future methane source potential of high latitude regions will depend, in part, upon the relative response of indigenous microbial communities (e.g. methanogens) to changing *in situ* environmental conditions (e.g. temperature and methanogenic substrate availability). Therefore, understanding the action and limitations of different trophic groups of anaerobic (and aerobic) microorganisms within cold environments could provide valuable information relating to region-specific latitudinal differences in microbial processes which ultimately control methane flux to the atmosphere [9].

Additionally, investigating the action and limitations of anaerobic communities in transiently and permanently cold environments may also help to enable the further development of more sustainable low temperature waste treatment systems. Specifically, optimisation of low temperature microbially mediated anaerobic waste treatment systems is hugely important in terms of developing more economic and environmentally sustainable means of treating waste. At present almost all commercial, and experimental, anaerobic waste treatment applications function at temperatures exceeding 18°C [10–11]. The majority operate at 30–40°C, or 50–60°C [10–11] and therefore have high associated energy requirements. Overwhelmingly, low temperature treatment systems (which are operated at temperatures below 20°C) are produced by inoculating reactors with mesophilic anaerobic sludge, which is then acclimatised to lower temperatures over extended periods of time (e.g. months to years) [11]. Bowen et al. [12] suggests that this strategy may ultimately be limited by the intrinsic biological properties of the biomass used. Therefore exploration of distinct biological properties of biomass endemic to distinct permanently or transiently cold environments, may provide an alternative route in the further development of stable low temperature anaerobic waste treatment systems with higher relative rates of activity.

Combined geochemical and culture independent studies of microbial communities which link methanogen activity to methanogen community structure in high latitude regions are still relatively scarce [13–16], and despite concerted efforts, few psychrophilic methanogens have been isolated from cold environments [14–20]. At present the most complete data relating to low temperature methanogen activity has been derived from thermally and geochemically stable permanently cold deep lake sediments [21–25]. These studies have shown that indigenous methanogenic communities are not exclusively adapted to low temperatures [21–22], and that the highest rates of methane production observed in incubated permanently cold sediments have been above *in situ* temperatures [21, 23–25]. However, some microcosm experiments, including low temperature pre-incubation of cold sediments, have identified functionally distinct communities of methanogens (including presumably obligate psychrophilic methanogens) within permafrost soils [26], and thermally stable deep lake sediments [27].

The main objective of this study was to gain an understanding of the broad effect of temperature (5 to 70°C) and methanogenic substrate amendment (i.e. acetate (10mM), methanol (10mM), or H_2_/CO_2_ (4:1 in headspace)), on the function and composition of methanogen communities within sediments from thermally dynamic high latitude environments affected by seasonal freeze-thaw (Høj et al. [28–29]). The findings reported have important implications for understanding the effect of climate change in natural environments. Moreover, the outcomes of this study can also be used to inform the potential use of inocula from permanently cold anoxic sediments to seed low-temperature anaerobic waste treatment systems.

## Materials and Methods

### Study site & sediment characteristics

Grab samples of high Arctic wetland sediment were taken from Solvatnet and Stuphallet, Ny-Ålesund, situated on the Brøgger peninsula Svalbard, Norway (78°50′N–11°30′E) during August 2007. These sites were the same as those sampled by Høj et al. [28–29] Sediment samples were stored under anoxic conditions until subsequent microcosm set up. The air temperature during sampling was ~5°C. The sediment surface temperature was 5°C. During the summer period in 2007 the absolute minimum and maximum air temperatures observed ranged from -1.4°C to 14.1°C (data obtained from the Norwegian Meteorological Institute), however the summer maximum can reach 16°C [30]. Livingstone and Lotter [31] determined that *in situ* lake temperatures (to a depth of 6 m) are similar to local atmospheric temperatures in high latitude environments. Therefore it is assumed in this study that samples obtained from the anoxic near surface sediment may exhibit similar thermal characteristics in the summer period to the recorded air temperature. Permission was obtained from the office of the Governor of Svalbard (Environmental Protection Department) to remove the samples and return them to the UK. The work did not involve any endangered species. Sediments were maintained at 4–5°C in an insulated cold box for transportation from Ny-Ålesund to Newcastle University and were stored unopened at 4–5°C until use (within 50 days of sampling). During sampling and storage there would be minimal oxygen incursion during this period and the sediment remained black in colour, indicating that iron sulphides in the sediment had not been oxidized and thus that the samples had remained anoxic.

Duplicate (~1 ml) sediment samples were dried to a constant weight at 80°C (to establish dry mass) and then heated to 550°C for 24 hrs in a muffle furnace (Carbolite AAF 110; Carbolite, Hope, UK). The weight loss was used to determine percentage organic carbon content expressed as percentage Loss on Ignition at 550°C (%LOI_550_). Pore water anion content was established using duplicate sub-samples of sediment which were centrifuged at 10,000 *g* for 10 minutes. Pore water fractions were diluted 500 fold in Milli-Q water, and analysed for sulphate (SO_4_^2-^) and chloride (Cl^-^) using a Dionex ICS-1000 Ion chromatograph, fitted with an AS14A column (length 4 m; Dionex, Camberley). The eluent comprised NaHCO_3_ (1 mM) and Na_2_CO_3_ (0.8 mM) with a flow rate of 1 ml/min. Peak areas were calibrated using standard solutions of SO_4_^2-^ (30 or 15 mg/l) and Cl^-^ (10 and or 7.5 mg/l) respectively. Salinity (S) was determined from Cl^-^ concentrations. Total cell numbers were determined using the method outlined in Gray et al. [32].

### Preparation and incubation of microcosms

Sulphate free anaerobic enrichment medium (1 ml) [33] and homogenised Arctic sediment (0.5–1 g), were added to serum bottles (12 ml, Wheaton) under anaerobic conditions. Bottles were closed with butyl rubber stoppers, crimp-sealed and purged with oxygen free nitrogen. Triplicate microcosms were amended with methanogenic substrates i.e. acetate (final concentration 10 mM), methanol (final concentration 10 mM), or H_2_/CO_2_ (4:1 in headspace), alongside triplicate unamended controls. Control microcosms treated with 2-bromoethane sulphonate (BES; 10 mM final concentration) were incubated at 30°C. Microcosms were incubated for a minimum of 28 days and up to 150 days, at temperatures of 5, 20, 30, 40, 50, 60, or 70°C. These temperatures were specifically chosen to enrich a wide range of thermally distinct communities of methanogens present in the original sediment, rather than to provide detailed temperature profiles of individual components of the methanogen community.

### Headspace methane analysis

Headspace methane was measured periodically by GC-FID using a Carlo ERBA HRGC 5160 fitted with a Chrompak PLOT fused silica capillary column (30 m x 0.32 mm) using helium as a carrier gas. CH_4_ was quantified on the basis of peak area and calibrated using CH_4_ standards (Scientific & Technical Gases Ltd, Newcastle-under-Lyme, UK). After 28 days Solvatnet microcosms incubated at 5 and 30°C were sacrificed for microbial community analysis. All higher temperature Solvatnet microcosms and all Stuphallet microcosms were incubated for a total of 150 days. Rates of methanogenesis were calculated from the linear accumulation of methane per gram of sediment dry mass (DM), and were compared statistically by one-way analysis of variance (ANOVA; Minitab 17, Minitab Ltd, Coventry, UK). The linear accumulation of methane over days 0–7 was used to calculate the Q_10_ value over a temperature range of 5 to 30°C [34].

### DNA extraction

Triplicate microcosms sacrificed for molecular analysis were stored at -20°C prior to DNA extraction. Single extractions were carried out on each of the triplicate sediment or microcosm slurries to create experimental replicates (~0.25 ml) using a FastPrep Ribolyser (Hybaid Ltd, Hampshire, UK) and a BIO 101 FastDNA Spin Kit (for soil; Q-BioGene, Cambridge, UK), according to the manufacturer’s instructions. DNA was quantified with a Nanodrop 2000 (Thermo Scientific, Wilmington, USA). PCR amplification of archaeal 16S rRNA genes was carried out for each of the triplicate samples for DGGE analysis and preparation of 16S rRNA gene clone libraries.

### PCR amplification of archaeal 16S rRNA genes for DGGE analysis

Archaeal 16S rRNA gene fragments were amplified using nested PCR. The first round of amplification was carried out with primers ARCH46f (5′-YTAAGCCATGCRAGT-3′) [35] and ARCH1017r (5′-GGCCATGCACCWCCTCTC-3′) [36]; resulting in a 971bp fragment (which was subsequently used in clone library construction, see below). For subsequent DGGE analysis a second round of PCR used ARCH344f-gc (5′CGCCCGCCGCGCGCGGCGGGCGGGGCGGGGGCACGGGGGGACGGGGHGCAGCAGGCGCGA-3′) [37] and UNIV522r (5′-GWATTACCGCGGCKGCTG-3′) [38] was used. This resulted in the production of a ~178 bp product (including GC-clamp). In all instances primers were provided by Fisher Scientific, Leicestershire, UK.

PCR reactions (50 μl) contained extracted DNA (~5–30 ng), primers (10 pmol of each), deoxynucleoside triphosphates (10 mM each), MgCl_2_ (1.5 mM), buffer (5 μl of 10 x stock) and Taq DNA polymerase (4U, Bioline, London, UK), and were carried out using an automated thermal cycler (G-storm GS1; GRI Ltd, Essex, UK) or a PCR Sprint (Hybaid Ltd, Hampshire, UK). The first PCR reaction comprised an initial denaturation step (94°C for 3 minutes), 30 cycles of denaturation (94°C for 1 minute), annealing (40°C for 1 minute) and extension (72°C for 1 minute) followed by a final extension of 72°C for 10 minutes.

When a second PCR reaction was used the first round products (1 μl) were used as a template. The denaturing step lasted for 3 minutes at 940078C, and was followed by 30 cycles of denaturation (940078C, 1minute), annealing (55°C, 1 minute), and extension (72°C, 1 minute). A final extension step was carried out at 72°C for 10 minutes. DGGE analysis of the archaeal 16S rRNA gene PCR products used a D-gene system (Bio-Rad, California, USA) as previously described [32]. Gels were stained with SYBR Gold (20 μl in 200 ml 1x TAE for 30 minutes, Sigma, Poole, UK) and viewed using a Fluor-S Multi Imager (Bio-Rad, California, USA). The BioNumerics software package (Applied Maths, Texas, USA) was used to produce normalized composite gels with reference to markers [39] comprising a mixture of PCR products from cloned 16S rRNA genes. These are individual clones from our own laboratory selected to provide a spread of bands on DGGE gels. Band positions were determined for individual community profiles. Cluster analysis was carried out to investigate the similarity within and between triplicate sample lanes on the basis of band presence/absence using the Dice coefficient (S_D_ = 2nAB/ [nA + nB]) where nAB is the number of bands shared between profile A and B and nA and nB are the total number of bands in profile A and B respectively. To determine the statistical significance of the similarity/difference between groups of samples, pairwise analysis of similarities (ANOSIM) was used to compare defined groups (i.e. amended triplicate microcosms treated with different methanogenic substrates).

### Archaeal 16S rRNA gene clone library construction

Based on DGGE analysis of triplicate community profiles, single representative microcosms and sediment samples were selected for 16S rRNA gene clone library construction. Specifically, clone libraries were generated from DNA extracted from the original sediment, from microcosms incubated at 5°C (including unamended, acetate amended and H_2_/CO_2_ amended microcosms), and at 30°C (acetate and H_2_/CO_2_ amended microcosms).

PCR products of partial 16S rRNA genes (after gel purification) were cloned using a TOPO TA cloning kit version U (Invitrogen), as described in the kit-supplied protocol. For each clone library 96 clones were randomly picked and grown in a 96-well plate in 100 μl LB medium containing 50 μg ml^-1^ ampicillin, at 37°C overnight. The presence of the correctly sized cloned insert was determined directly on the cultured cells using vector-targeted primers. Clones were preserved in glycerol (final concentration ~15% v/v) and stored at -80°C. PCR products from all clones with the correctly sized insert were digested for ARDRA analysis with the restriction enzymes *Hae*II and *Hha*I (Takara BIO INC. Otsu, Shiga, Japan).

The reaction mix consisted of 5 units of each enzyme, 1x M buffer (100 mM Tris-HCl pH7.5, 100 mM MgCl_2_, 10 mM Dithiothreitol, 500mM NaCl), and 2.5 μl PCR product. Reactions were carried out at 37°C for 60 minutes and at 65°C for 20 minutes for *Hae*II and *Hha*I respectively. Patterns were visualized by running the digested DNA in a 3% agarose gel at 100 V for 120 min. DNA in the gel was subsequently stained for 45 min in 0.5 μg/ml ethidium bromide solution. Fingerprint patterns were visualized as for ethidium bromide stained agarose gels.

For each distinct Amplified Ribosomal DNA Restriction Analysis (ARDRA) pattern (S1 Table) between 2 and 8 representative clones were sequenced from each of the libraries. The total number of sequences acquired was 109 (S1 Table). Sequences were checked and assembled using BioEdit [40]. Assembled sequences were ca. 890 bp in length. A single chimeric sequence was identified using Mallard [41] and excluded from further analysis. When sequences were identical only one was deposited within the GenBank database (44 sequences in total; FR845726- FR845769).

Phylogenetic distance analysis was conducted using the Jukes-Cantor correction for multiple substitutions at a single site and the neighbour joining method, based on a total of 854 positions using the MEGA 6 software package [42]. Sequences were compared to sequences from environmental samples and cultured organisms in the EMBL-GenBank database and the SILVA database (http://www.arb-silva.de).

## Results

### Sediment characteristics

Sediments from Solvatnet and Stuphallet wetlands were distinct in terms of physical structure, chemical characteristics and total cell numbers. Solvatnet sediment comprised partially degraded fibrous plant material while Stuphallet sediment comprised fine gravel. Extracted pore water indicated that Solvatnet sediment had low salinity and sulphate levels consistent with a freshwater origin (salinity 0.075 ± 0.007‰, sulphate levels below the detection limit of 0.0014 mM), while Stuphallet sediment had a higher salinity and sulphate level (6.504 ± 0.757‰ salinity and 0.431 ± 0.022 mM sulphate). The organic matter content of Solvatnet sediment was high (60.96 ± 3.79%), as reported previously in studies of the area [28], while Stuphallet sediment organic matter content was far lower (13.24 ± 2.29%). Total cell numbers in Solvatnet sediment were higher than in Stuphallet sediments (1.43 ± 0.04 x10^9^ and 3.21 ± 0.09 x10^8^ cells/g^-1^ dry sediment respectively) and were found to be significantly different (p = 0.001, T Test for independent means, Minitab 17).

### Methanogenesis in Arctic sediment microcosms

**Fig 1:**
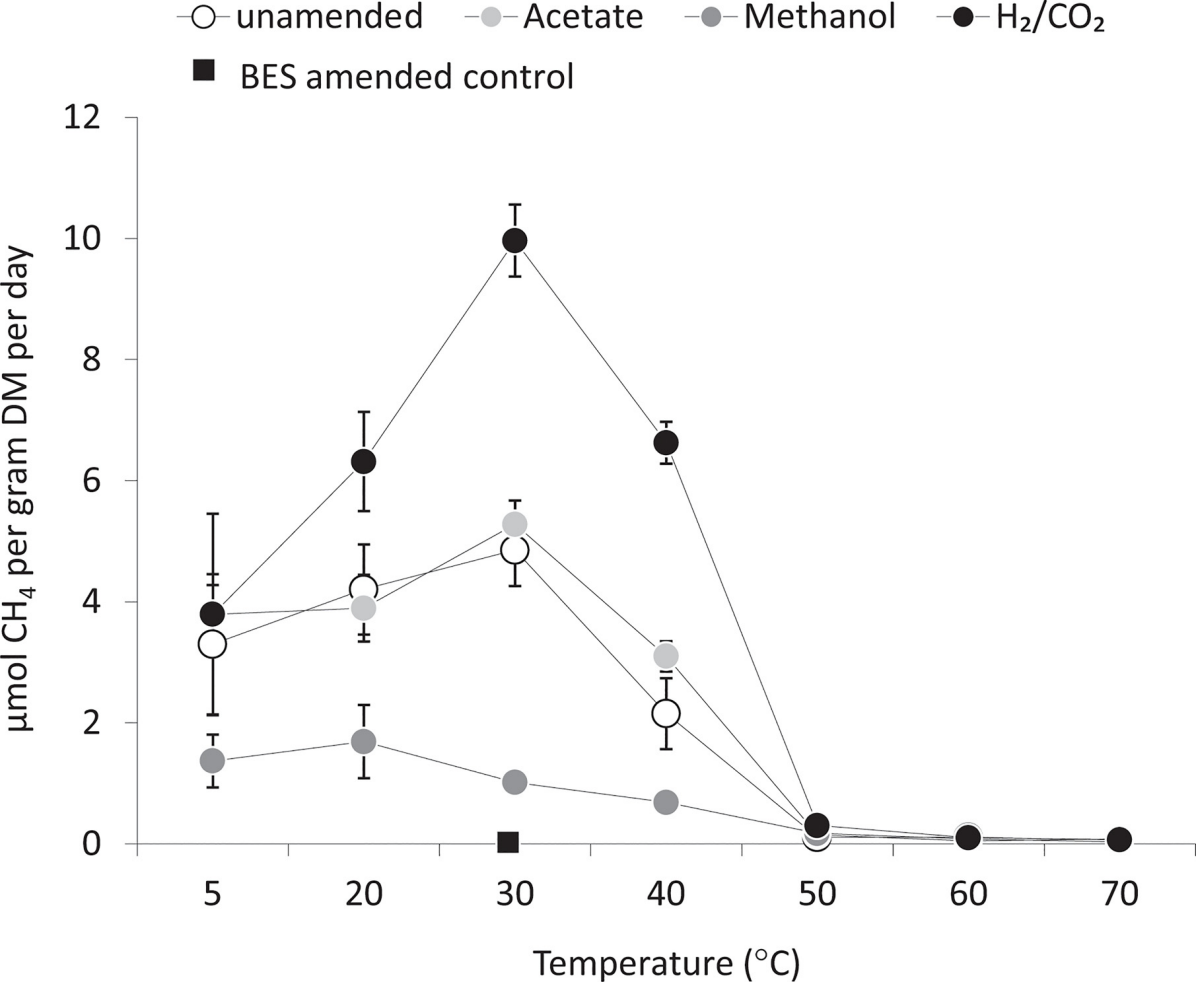
Methane production rates over the incubation temperature range of 5 to 70°C from day 0 to 7 with or without methanogenic substrate addition. Methane production in BES controls was always very low (maximum 0.0107 ± 0.001 μmol CH_4_ g^-1^ DM d^-1^ ± SE, n = 3).

Methane production (Fig 1) was not detected during a 150 day incubation period in any of the Stuphallet sediment microcosms (5–70°C) and in any of the Solvatnet sediment microcosms incubated at temperatures above 40°C (Fig 1). Methane production was exceptionally low in all the BES inhibited controls (maximum rate 0.0107 μmol CH_4_ g^-1^ DM d^-1^; (± 0.001, n = 3)). In contrast, methane production was detected within 24 hours in the Solvatnet sediment microcosms incubated at temperatures between 5 and 40^°^C. In these microcosms methane accumulated progressively for the first 21 days in both substrate amended and substrate unamended microcosms. A comparison of methane production rates at different temperatures was made for different trophic groups of methanogens over the time period of 0–7 day’s incubation (Fig 1). Methane production rates were dependent on methanogenic substrate addition and incubation temperature (Fig 1). Optimum methane production rates were observed at 30^°^C (unamended microcosms 4.85 ± 0.59 μmol CH_4_ g^-1^ DM d^-1^; acetate amended microcosms 5.28 ± 0.40 μmol CH_4_ g^-1^ DM d^-1^; H_2_/CO_2_ amended microcosms 9.96 ± 0.60 μmol CH_4_ g^-1^ DM d^-1^), in all but methanol amended microcosms. In methanol amended microcosms methane production rates were significantly lower than all other microcosms including unamended controls (the maximum methane production rate at 20°C was 1.96 ± 0.61 μmol CH_4_ g^-1^ DM d^-1^). This indicated an inhibitory of effect of methanol addition regardless of incubation temperature (one-way ANOVA, p>0.05). This inhibitory effect may be related to methanol toxicity. However, the concentration of methanol amendment used in this study is comparable to previous work on methylotrophic methanogenesis, including a parallel study investigating methanogenesis in temperate sediments [43], and previous studies of high latitude sediments [29]. Additionally, the concentration of methanol used is within the range used in growth media for pure cultures of methylotrophic methanogens (e.g. *Methanolobus psychrophilus* R15) [44–45].

Comparable rates of methane production (i.e. from 3.30 ± 1.16 to 3.80 ± 1.66 μmol CH_4_ g^-1^ DM d^-1^) were seen in all microcosms incubated at 5°C (one-way ANOVA, p = 0.358). In contrast at higher temperatures methane production rates in H_2_/CO_2_ amended microcosms (9.97 ± 0.60 μmol CH_4_ g^-1^ DM d^-1^) were significantly higher than methane production rates in unamended microcosms (4.85 ± 0.74 μmol CH_4_ g^-1^ DM d^-1^; one-way ANOVA, 30 and 40°C incubations, p<0.01). At all temperatures methane production rates in acetate-amended microcosms were not significantly different from unamended controls (one-way ANOVA, 5, 20, 30, and 40°C incubations, p>0.05) (Fig 1).

Calculation of the temperature coefficient (Q_10_), indicated that in all but methanol amended microcosms (where the Q_10_ value was 0.93 (± 0.23)), increasing temperature played a clear role in enhancing methane production, and that substrate amendment did not have a significant effect on Q_10_ values (one-way ANOVA, p = 0.432) when comparing unamended (Q_10_ = 1.33 ± 0.34), acetate amended (Q_10_ = 1.15 ± 0.30) and H_2_/CO_2_ amended (Q_10_ = 1.75 ± 0.41) microcosms.

### Effect of temperature on archaeal community structure

**Fig 2:**
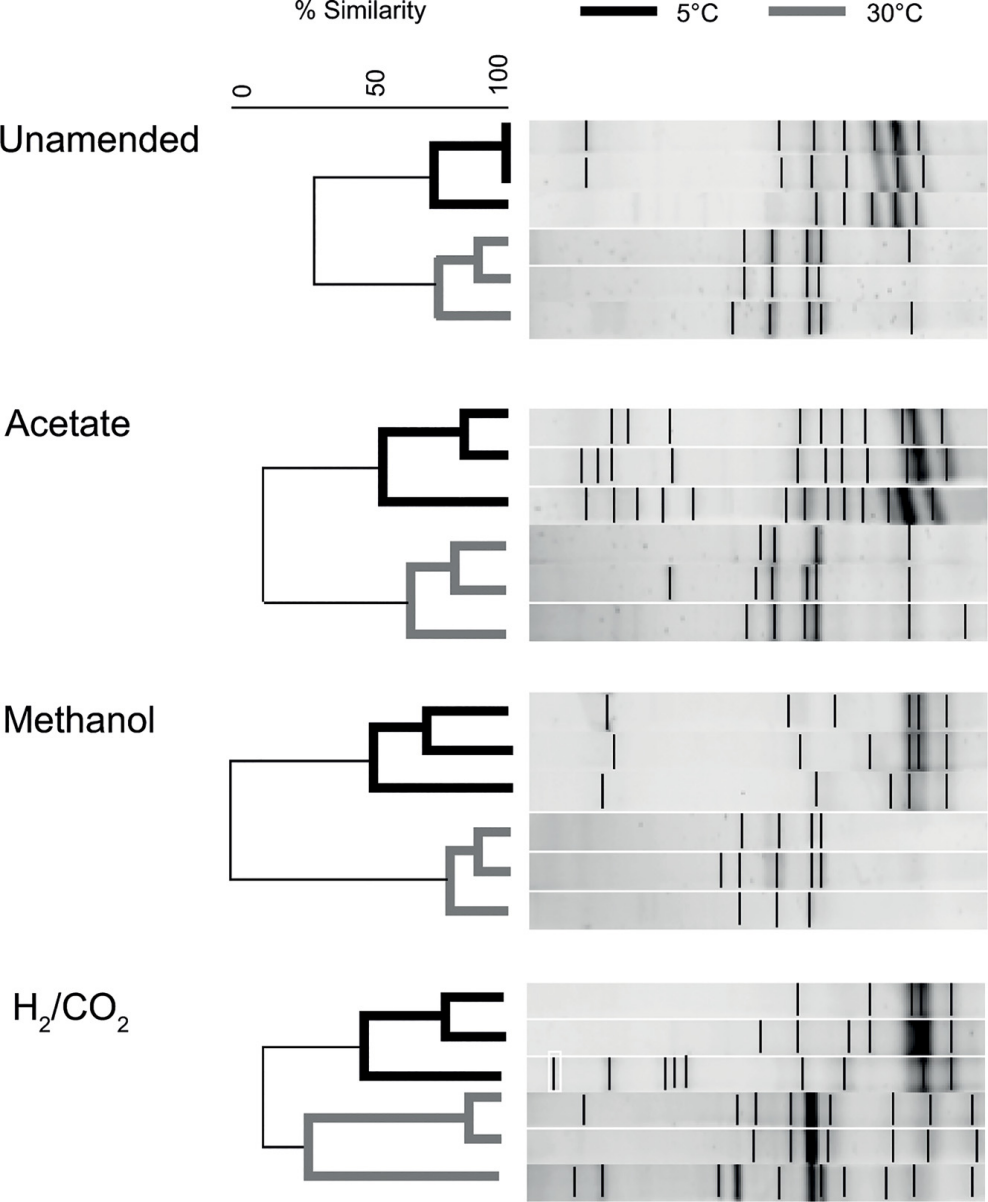
Cluster analysis of archaeal 16S rRNA gene DGGE profiles. Data for 5 and 30°C microcosms (black and grey respectively) are shown for substrate amended and unamended microcosms.

Comparison of DGGE profiles from substrate amended microcosms incubated at 5°C and 30°C indicated a clear shift in methanogen community structure in response to incubation temperature (Fig 2) but not substrate amendment. ANOSIM analysis, a rank based method of analysis of similarities within and between groups was used to compare the methanogen community structures. This analysis provides an R statistic (global R value) and P value (significance value). When R = 0 there is no separation between different groups of data, as R increases towards 1 the groups become increasingly more distinct. In this study ANOSIM analysis indicated that temperature (global R value of 0.834; p = 0.001) was responsible for structuring the methanogen community but that substrate amendment was only borderline significant (global R value = 0.135; p = 0.062).

**Fig 3:**
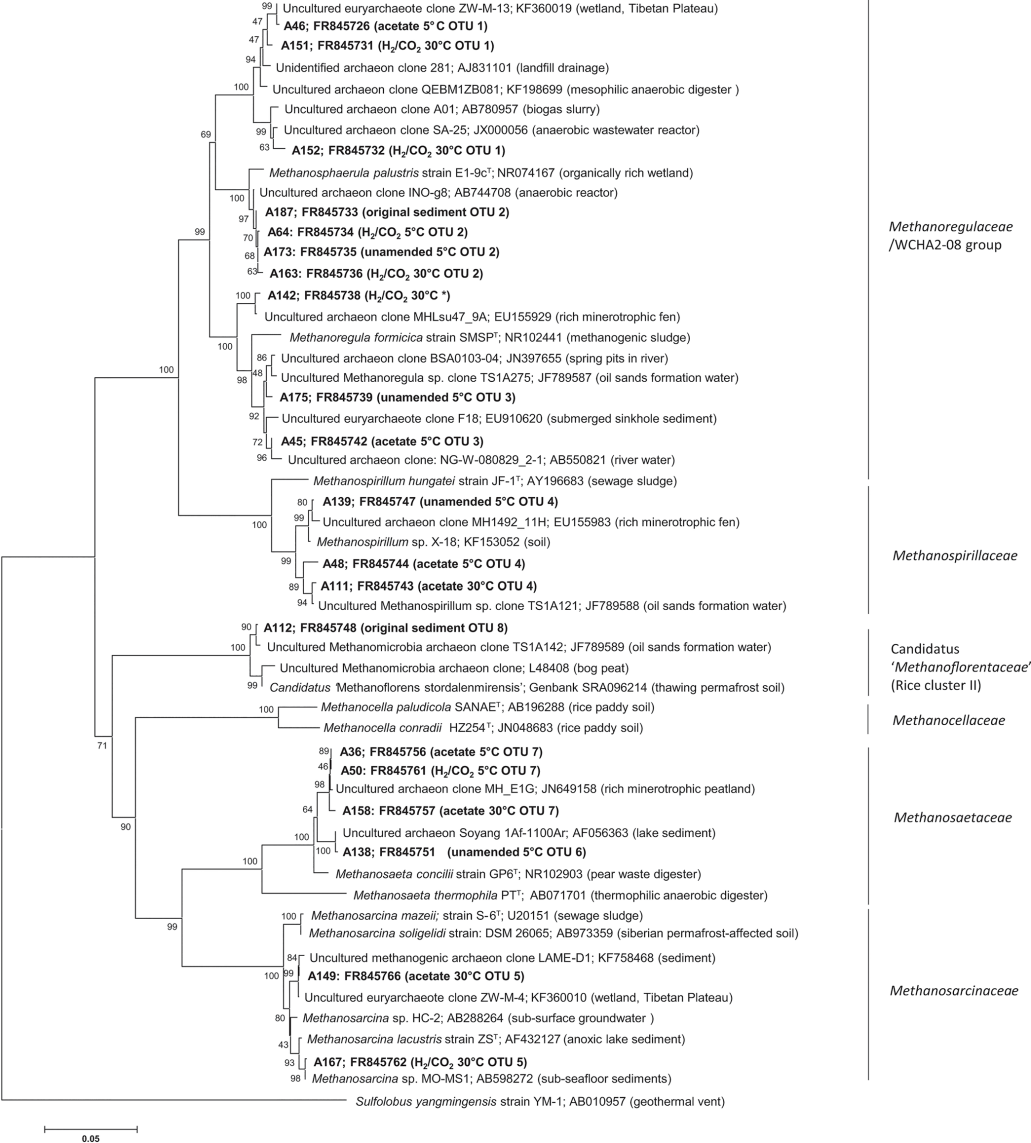
Phylogenetic tree of a selection of archaeal partial 16S rRNA sequences obtained in this study (shown in bold with sample origin and OTU assignments based on ARDRA analysis, see S1 Table). Taxonomic assignments for all sequences (including those not shown in this tree) were made using the SILVA database http://www.arb-silva.de. All sequences were assigned to the Class *Methanomicrobia* and designated (see brackets) as 5 separate families in the class *Methanomicrobia* i.e. *Methanoregulaceae*/WCHA2-08 and *Methanospirillaceae* (order *Methanomicrobiales*); *Methanoflorentaceae* (order *Methanocellales*); *Methanosaetaceae* and *Methansarcinaceae* (order *Methanosarcinales*). *Clone sequence A142 was classified based on ARDRA analysis as OTU 2 but was found to be more distantly related than all other representative OTU 2 sequences albeit clustering within the same family-level group *Methanoregulaceae*/WCHA2-08.

**Fig 4:**
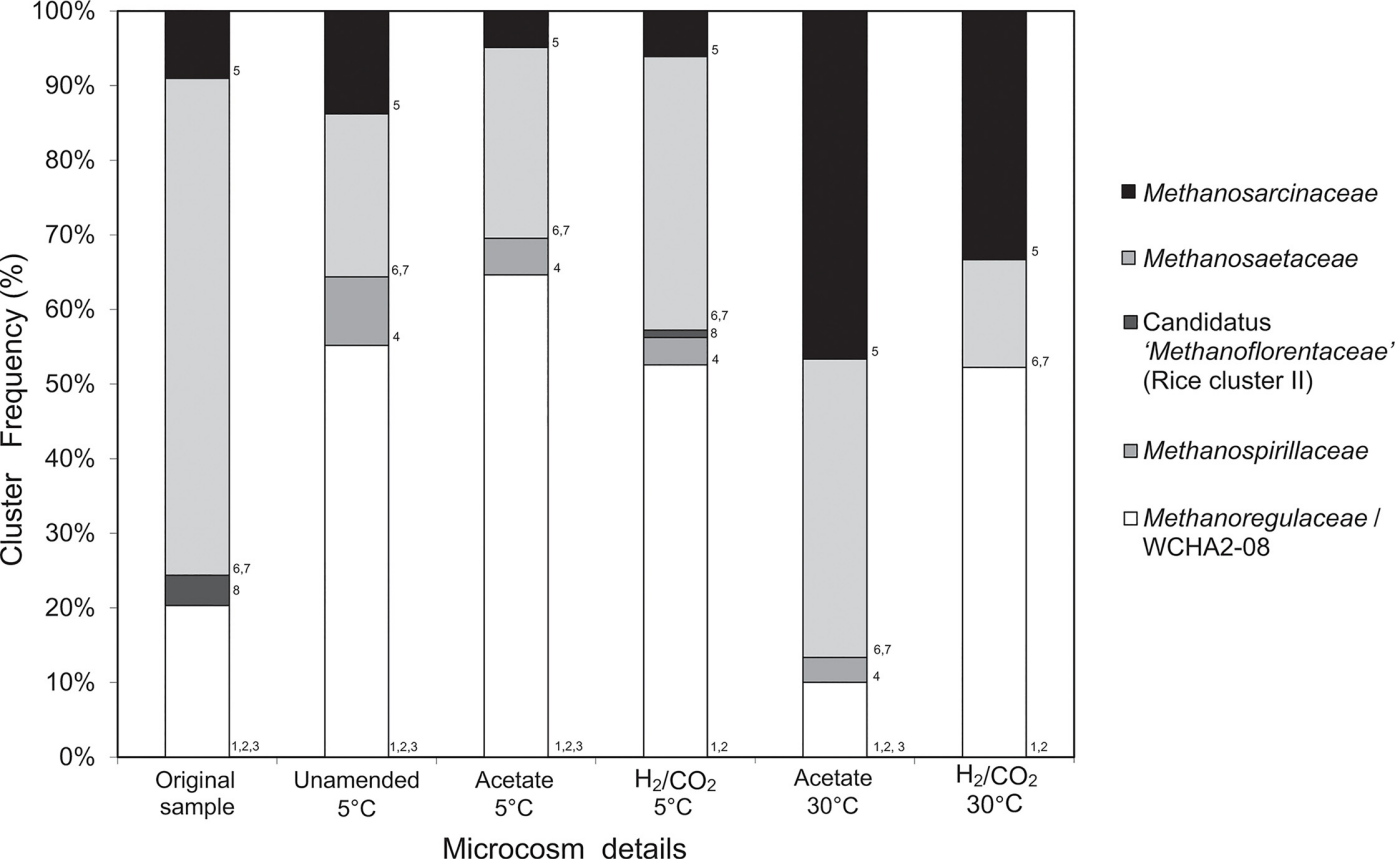
Relative abundance (%) of archaeal family level groups within each of the microcosms and the original sediment sample based on the frequency of the 8 distinct ARDRA patterns identified in the 16S rRNA gene clone libraries. These 8 ARDRA patterns were resolved into 5 different family level groups based on the phylogenetic assignments of representative clone sequences (see Fig 3). The numbers adjacent to the columns indicate which ARDRA patterns contributed to the groups in individual microcosms.

Clone libraries were prepared from the original sediment, unamended 5°C microcosms, H_2_/CO_2_ 5°C microcosms, acetate 5°C microcosms, H_2_/CO_2_ 30°C microcosms, and acetate 30°C microcosms. ARDRA analysis of clone libraries resulted in 8 distinct patterns (OTUs) across all clone libraries (S1 Table). Analysis of 16S rRNA sequences from clone libraries resolved these 8 distinct OTUs into 5 family-level groups. Sequences used in the phylogenetic analysis are representative of these groups (Fig 3). The frequencies of these 5 family level groups (and constituent OTUs) are represented in Fig 4. The frequencies of OTUs for each library are provided in supplementary material (S1 Table).

In all instances cloned 16S rRNA gene sequences were most closely related to known cultured or uncultured methanogens belonging to the Class *Methanomicrobia*, orders *Methanomicrobiales*, *Methanocellales* or *Methanosarcinales*, and displayed high sequence identity with database sequences, regardless of microcosm incubation temperature or substrate amendment, with high bootstrap values supporting their phylogenetic assignments (Fig 3). Sequences from the order *Methanomicrobiales* comprised two family level groupings *Methanoregulaceae*/WCHA2-08 group (OTU 1, 2, 3) and the *Methanospirillaceae* group (OTU 4), while the order *Methanosarcinales* was represented by *Methanosarcinaceae* (OTU 5) and *Methanosaetaceae* (OTU 6,7) family level groupings, and the order *Methanocellales* was represented by Candidatus ‘*Methanoflorentaceae*’ (Rice cluster II) [46] (OTU 8) (Fig 3). It has recently been proposed by Mondav et al. [46] that the family level group designated as Rice Cluster II (order *Methanomicrobiales*) in the SILVA database, forms a new family, Candidatus ‘*Methanoflorentaceae*’ (order *Methanocellales*). The proposal of Candidatus ‘*Methanoflorentaceae*’ was based on analysis of concatenated protein sequences derived from 104 conserved archaeal marker genes which more clearly demarcates this group’s phylogenetic position, which is uncertain based on analysis of the SSU rRNA gene alone. This uncertainty is clear in the 16S rRNA- based tree presented here which included other sequences from the order *Methanocellales* (*Methanocellaceae*) which are not monophyletic with the sequences from Candidatus ‘*Methanoflorentaceae*’ (Fig 3).

From clone frequency distributions (Fig 4), clear differences existed between clone libraries from the original sediment and incubated microcosms; microcosms incubated at 5 and 30°C; and between clone libraries from microcosms incubated at 30°C with different substrates. For instance, within the original sediment *Methanosaetaceae* 16S rRNA genes made up 66% of the clone library, with 20% from *Methanoregulaceae*/WCHA2-08, and smaller proportions of *Methanosarcinaceae* (9%) and Candidatus ‘*Methanoflorentaceae*’ (Rice cluster II; 4%). After low temperature incubation clone libraries had a very similar clone frequency structure regardless of methanogenic substrate amendment (Fig 4). Specifically, all 5°C clone libraries were dominated by genes from *Methanoregulaceae*/WCHA2-08 (53–65%) with lower representation of sequences from *Methanosaetaceae* (22–37%) and *Methanosarcinaceae* (5–14%), and a low representation of *Methanospirillaceae* sequences (4–9%) (Fig 4). In the low temperature H_2_/CO_2_-amended microcosms Candidatus ‘*Methanoflorentaceae*’ (Rice cluster II) was detected at a very low frequency (1%) (Fig 4).

In contrast, when incubated at higher temperatures (30°C) methanogenic substrate amendment resulted in distinct changes in clone frequency distributions. Specifically, when microcosms were amended with acetate *Methanosarcinaceae* (47%) and *Methanosaetaceae* (40%) made up the major part of the community, while *Methanoregulaceae*/WCHA2-08 (10%) and *Methanospirillaceae* (3%) were minor components (Fig 4). When microcosms were amended with H_2_/CO_2_ the community was in contrast dominated by *Methanoregulaceae*/WCHA2-08 (52%), followed by *Methanosarcinaceae* (33%), and *Methanosaetaceae* (15%) (Fig 4).

The 5 family level lineages identified in this study were most closely related to known methanogens including psychrotolerant methanogens isolated from permanently or transiently cold environments, and mesophilic and indeed thermophilic methanogens previously isolated from natural or engineered systems (Fig 3). The original sediment was dominated by the *Methanosaetaceae* group (66%) with clone sequences most similar to *Methanosaeta concilii* strain GP6 (T) a moderate thermophile isolated originally from a pear waste digester. *Methanosaeta concilii* strain GP6 has a temperature range of 10–45°C and T_opt_ of between 35 and 40°C [47]. In all 5°C incubated microcosms and 30°C H_2_/CO_2_ amended microcosms sequences for the dominant *Methanoregulaceae*/WCHA2-08 group (up to 65%) were most closely related to *Methanosphaerula palustris*, a recently cultured, mesophilic, hydrogenotrophic methanogen isolated from a minerotrophic fen [48–49]. They were also closely related to *Methanoregula formicica* strain SMSP (T) a mesophile originally derived from methanogenic sludge used to treat brewery effluent. *Methanoregula formicica* strain SMSP has a growth range of 10–40°C and a T_opt_ of 30–33°C [50]. In contrast in 30°C acetate and H_2_/CO_2_ amended microcosms the *Methanosarcinaceae* group sequences were most closely related to *Methanosarcina lacustris* strain ZS (T) a psychrotolerant methanogen (which can utilise methanol, mono, di, trimethylamine and H_2_/CO_2_ for methanogenesis) and has a growth range of 1–35°C and T_opt_ of 25°C [20]. The *Methanospirillaceae* group was detected in all 5°C microcosms, and in the 30°C acetate-amended microcosms. The most closely related cultured microorganism within this cluster was *Methanospirillum hungatei* strain JF-1, a mesophile previously isolated from sewage sludge, T_opt_ 37°C. In the original sediment and 5°C H_2_/CO_2_ amended microcosms a small proportion of sequences was affiliated to the Candidatus ‘*Methanoflorentaceae*’ (Rice cluster II) group, and most closely related to Candidatus ‘*Methanoflorens stordalenmirensis’*. Recent metagenomic analysis has suggested that Candidatus ‘*Methanoflorens stordalenmirensis’* is a hydrogenotrophic methanogen. Interestingly, it has been postulated that Candidatus ‘*Methanoflorens stordalenmirensis’* may be a key mediator of methane-based positive feedback climate warming in cold regions undergoing permafrost thawing [46].

## Discussion

Although cold, high latitude regions are important contributors to the global atmospheric methane flux, a great deal of uncertainty surrounds the potential response of indigenous microbial communities to changing *in situ* environmental conditions. Previous studies have shown that temperature [21–22, 51], and substrate availability [52], are key drivers of microbial processes in permanently and transiently cold environments. In this study we have gained a broad understanding of the effect that temperature (5 to 70°C), and methanogenic substrate amendment, can have on the function and composition of the methanogen community within sediments from thermally dynamic high latitude environments affected by seasonal freeze-thaw.

### Methanogen function and methanogen community structure in response to temperature

Previous studies of Arctic, sub-Arctic and Antarctic regions have suggested that microbial community structure and function are relatively homogenous, both spatially and temporally, within geographically disparate anoxic soils and sediments [14, 15, 22, 27]. In agreement with this, previous studies of the sites investigated (Solvatnet and Stuphallet) have suggested that although sediment characteristics are both physically and chemically distinct, total cell numbers (biomass), methanogenic potential (function) and methanogen community structure were spatially and temporally similar [13, 28]. In contrast, the current study indicates that in addition to the clear distinctions which exist between the physical and chemical characteristics of the two sites, total cell numbers, and methanogenic potential are also distinct. Specifically, although methanogenesis was detected in the Solvatnet sediment microcosms (within 24 hrs of incubation), it was not detected in Stuphallet sediment microcosms, even following an extended incubation period of 150 days. The discrepancy between the findings of this study and previous studies of these sites [13, 28] may simply reflect an undefined spatial and/or temporal heterogeneity within the sediment. This serves to highlight a wider issue, namely, that clarification of microbial process; heterogeneity, and functional characteristics, within climatically sensitive cold environments could be pivotal in developing a clear understanding of the effect that future climate change will have on these regions. The recent work of Yvon-Durocher et al. [51], has indeed indicated that when synthesised from large scale meta-data sets, microcosm level studies (such as this one) can provide relevant insights into ecosystem level function in response to key factors (e.g. temperature, substrate, nutrients). Therefore using parameterisation derived from microcosm based approaches to represent microbial function within Earth System Models (ESMs) could inform more accurate modelling of CH_4_ fluxes under different climatic scenarios. Various suggestions have been put forward as means to practically incorporate this objective e.g. using microbial community response [53], microbial biomass [54], or Q_10_ values [55].

In the present study temperature is clearly the dominant factor determining methanogen community function (Fig 1) and methanogen community structure (ANOSIM analysis of data from Fig 2, global R value of 0.834; p = 0.001). In agreement with previous studies, methane production is possible at temperatures within, and far above, the environmental *in situ* temperature range (of between -1.4 and 14.1°C). Specifically the T_opt_ was between 30 and 40°C, with a range of at least 5 to <50°C. However, in contrast to the study of Nozhevnikova et al. [21], where modal ranges were clear, in this study no distinction could be made between methanogenic communities based on methane production rates and thermal ranges alone (Fig 1). The maximum rates of methanogenesis in low temperature (5°C) microcosms (3.5 μmol CH_4_ g^-1^ DM d^-1^) were comparable to previous microcosm studies of permanently cold deep lake sediments (of 7.27 μmol CH_4_ g^-1^ DM d^-1^) [21] but were significantly higher than comparable microcosm studies of temperate sediments (maximum rate 0.036 μmol CH_4_ g^-1^ DM d^-1^; one-way ANOVA, based on site, p< 0.001) [43], and were far higher than methane production rates noted in microcosm studies of low temperature waste treatment systems using acclimatised mesophilic sludge (maximum rate 0.23 μmol CH_4_ g^-1^ DM d^-1^) [12].

We used the temperature coefficient (Q_10_), to draw further comparison of our data with prior studies of methanogen function in polar, temperate and tropical environments. With the exception of the recent study of Treat et al. [34] where the median Q_10_ for methane production of pan-arctic sites was 1.19, the Q_10_ values of this study are low in comparison to existing studies of methanogenesis in polar [55,56], temperate [43, 55, 57–59], and tropical environments [56] where the Q_10_ range was typically between 2.7 to 5, and was generally enhanced by direct or indirect methanogenic substrate amendment (e.g. Q_10_ reaching between 10 to 40 in [43, 56, 60, 61]), or by the organic carbon ‘quality’ within the sediment [55].

The comparatively low Q_10_ values (over the temperature range of 5–30°C) determined in this study can be reasonably explained by the presence of low temperature adapted methanogens in these sediments which serve to reduce the measured differences in methanogenic rates as a function of temperature. Such low temperature adapted methanogens (over the short term at least) are apparently absent from temperate sediments where Q_10_ values in the range 2.96–9.75 have been observed [43]. On the basis of these observed differences, Q_10_ values may allow rapid investigation of functional differences within and between transiently and permanently cold environments. This would provide a favourable means of representing microbial function within Earth System Models (ESMs).

Additionally, the use of Q_10_ values could be valuable in identifying the intrinsic biological properties of biomass endemic to distinct permanently or transiently cold environments for the subsequent development of low temperature waste treatment systems. For example, in this role the ability of low temperature microorganisms to sustain relatively higher levels of activity at lower temperatures, due to the low temperature adaptation of their proteins and intracellular solutes in terms of activity and stability [25] may allow the development of stable, lower temperature (<20°C) anaerobic waste treatment systems with higher relative rates of biogas production, therefore being more economically and environmentally viable.

The methanogens most phylogenetically similar to those from the 5 representative lineages identified in this study included known psychrotolerant methanogens [20], mesophiles isolated from natural and engineered environments [48–50, 62], as well as moderate thermophiles [47]. Although, under low temperature incubation conditions the predominant methanogens in our study were clearly very similar at the 16S rRNA sequence level to mesophilic organisms such as *Methanosphaerula palustris* (i.e. up to 99% sequence identity at 5°C; Fig 3; S1 Table, OTU 2), they are phenotypically distinct with respect to thermal tolerance. Simankova et al. [19–20], previously noted that a number of methanogens isolated from natural or engineered cold environments have thermal limits of activity which are much lower (10 to 15°C lower) than the most closely phylogenetically related species, despite sharing very high 16S rRNA sequence identity (99.6 to 99.9%). This serves to highlight that phylogeny alone cannot be used as a predictor of microbial function or the limits of function, in low temperature environments. This conclusion emphasises the need for the isolation of more psychrophilic and psychrotolerant methanogens [19–20]. We propose that using combined approaches to study biogeochemical processes may be more favourable in defining the relationships that exist between microbial community structure and function in distinct environments, and that the synthesis of the resulting data will allow more meaningful interpretation at the ecosystem level [63], or in application to low temperature engineered biological systems [12].

### Methanogen function and methanogen community structure in response to methanogenic substrate amendment

It is often suggested that acetate is the most important methanogenic substrate in low temperature environments. Specifically, because at low temperatures syntrophic activity is reduced, and homoacetogenic activity is enhanced, favouring acetoclastic methanogenesis, and therefore enhancing the contribution of acetoclastic methanogenesis to total methane production [27, 64–66]. This has been supported by studies of 16S rRNA and methyl coenzyme M reductase (*mrcA*) genes in northern bogs and mires (where a large proportion of sequences recovered had high homology with *mcrA* from known acetoclastic methanogens i.e. > 66% [14, 52]. In agreement with this wider contextual literature on cold environments, the methanogen community in the native sediment studied here was dominated by acetoclastic methanogens (*Methanosaetaceae*), which made up over 66% of clone library sequences, while metabolically more versatile methanogens and hydrogenotrophic methanogens made up a lower proportion of the community (Fig 4). In contrast, previous studies of this specific site determined that communities of both acetoclastic and hydrogenotrophic methanogens were present in the sediment, and that regardless of season or sediment depth acetoclasts made up only ~25% of the methanogen community [28–29]. Furthermore, in contrast with previous studies of this site [28–29], we have shown that the methanogen community structure in the native sediment was reproducibly distinct from the methanogen communities that developed in all 5°C and 30°C microcosm incubation experiments.

It was anticipated that methanogenic substrate addition would enhance methane production [67–68], and that the methanogen community structure would be influenced, in part by substrate amendment. Interestingly, at low temperatures when exogenous methanogenic substrate was added (acetate or H_2_/CO_2_) this had no significant effect on the rate of methanogenesis (5 and 20°C), or on the methanogen community structure (5°C), which was however distinct from the original sediment. This indicated that in this organic carbon rich, low temperature system, harbouring psychrophilic or psychrotrophic methanogens, availability of methanogenic substrates does not limit methanogen activity. Stimulation of methanogenesis by addition of substrate at 30°C but not at 5°C may reflect differences in the maximal rates of methanogenesis possible at these temperatures. At the low rates of methanogenesis observed at 5°C the system may already be saturated with respect to available substrates and thus addition of exogenous substrate has no stimulatory effect. At 30°C the maximum rate of methanogenesis may not be sustainable on the basis of the levels of endogenous methanogenic substrate generation and therefore addition of exogenous substrate has a stimulatory effect. These results may indicate that in transiently cold environments, methanogen communities can rapidly respond to moderate short term increases in temperature but not necessarily to the organic carbon released from previously frozen sediment by freeze-thawing. However, if temperatures rise further carbon inputs will have a greater influence on methane production and methanogen community structure. Understanding the action and limitations of anaerobic microorganisms within cold environments may provide information which can be used in defining region-specific differences in the microbial processes; which ultimately control methane flux to the atmosphere.

## Conclusion & Future Work

In summary we found that Solvatnet and Stuphallet sediments were distinct in terms of physical and chemical characteristics as well as methanogenic potential. Within Solvatnet sediment microcosms, temperature was the dominant factor determining rates of methanogenesis and structuring the methanogen community.

We suggest that the transiently cold sediment studied may provide a distinct low temperature function which is not detected in short term studies of temperate sediments, and from low temperature acclimatised waste water treatment systems, and that utilisation of the Q_10_ value may be useful in developing ESMs, and in exploring the potential of complex natural low temperature systems as potential ‘seeds’ for low temperature waste treatment systems.

Clearly this study provides a snap-shot of a single process within a complex environment which may change both spatially and temporally. However, recent studies [51, 69], clearly highlight the value of microcosm based approaches and the potential for synthesis of data from different studies to provide greater insight into the relationships which control methane production potential and methanogen community structure in cold environments.

## Supporting Information

S1 Table ARDRA based OTU classification. The number of positive clones produced from each sample is provided along with the frequency of OTUs for each clone library based on ARDRA analysis. OTUs were subsequently resolved into 5 family- level groups, based on analysis of 16S rRNA sequences from clone libraries i.e. *Methanoregulaceae*/WCHA2-08 group (OTU 1, 2, 3), the *Methanospirillaceae* group (OTU 4), *Methanosarcinaceae* group (OTU 5), *Methanosaetaceae* group (OTU 6, 7) and Candidatus ‘*Methanoflorentaceae*’ (Rice cluster II) group (OTU 8). *One positive clone had a unique ARDRA pattern but when sequence for this clone was obtained and analysed using Mallard [41] it was identified as a possible chimera and omitted from further analysis.
(PDF)
