# Supplementary material for: Response of Methanogens in Arctic Sediments to Temperature and Methanogenic Substrate Availability
Source: PLoS One. 2015 Jun 17;10(6):e0129733. doi: 10.1371/journal.pone.0129733 (PMC4471053; doi:10.1371/journal.pone.0129733)
Supplement: S1 Table — The number of positive clones produced from each sample is provided along with the frequency of OTUs for each clone library based on ARDRA analysis. OTUs were subsequently resolved into 5 family- level groups, based on analysis of 16S rRNA sequences from clone libraries i.e. Methanoregulaceae/WCHA2-08 group (OTU 1, 2, 3), the Methanospirillaceae group (OTU 4), Methanosarcinaceae group (OTU 5), Methanosaetaceae group (OTU 6, 7) and Candidatus ‘Methanoflorentaceae’ (Rice cluster II) group (OTU 8). *One positive clone had a unique ARDRA pattern but when sequence for this clone was obtained and analysed using Mallard [41] it was identified as a possible chimera and omitted from further analysis. (PDF) [file pone.0129733.s001.pdf]

Table S1. ARDRA based OTU classification.

Sample	# clones in library	ARDRA-based OTU (number of clones)								# clones sequenced
		Methanoregulaceae			*Methanospirillaceae*	*Methanosarcinaceae*	*Methanosaetaceae*		*Candidatus* 'Methanoflorentaceae'	
		OTU 1	OTU 2	OTU 3	OTU 4	OTU 5	OTU 6	OTU 7	OTU 8	
Sediment	89	5	9	4	0	8	34	25	4	22
Unamended 5°C	87	2	42	4	8	12	8	11	0	22
Acetate 5°C	82	5	43	5	4	4	9	12	0	16
H_2/CO_2 5°C	81*	2	41	0	3	5	14	15	1	16
Acetate 30°C	60	2	3	1	2	28	2	22	0	16
H_2/CO_2 30°C	90	2	45	0	0	30	3	10	0	17
